# Mechanisms of constipation alleviation by *Lacticaseibacillus paracasei* BGI-N2: insights from genes to phenotypes

**DOI:** 10.1186/s12866-026-04831-0

**Published:** 2026-02-20

**Authors:** Xing Rao, Qiang Luo, Wanting Wei, Benliang Wei, Xinyu Yang, Yanhong Liu, Yangfeng Wen, Zhihui Ma, Zhinan Wu, Haifeng Zhang, Liang Xiao, Yiyi Zhong, Yuanqiang Zou

**Affiliations:** 1 State Key Laboratory of Genome and Multi-omics Technologies, BGI Research, Shenzhen 518083, China; 2 College of Life Science, University of Chinese Academy of Sciences, Beijing 100049, China; 3 BGI Precision Nutrition, Shenzhen, 518083 China; 4 BGI Research, Shenzhen, 518083 China; 5 Shenzhen Key Laboratory of Human commensal microorganisms and Health Research, BGI Research, Shenzhen, 518083 China

**Keywords:** Constipation, *Lacticaseibacillusparacasei*, Complete genome, Zebrafish, 5-hydroxytryptamine

## Abstract

The global prevalence of constipation has been increasing in recent years, posing an urgent need for safer and more effective therapeutic strategies. Given the favorable safety profile and beneficial effects on gut health, probiotics have emerged as a promising means for improving constipation. This study investigated the probiotic potential of *Lacticaseibacillus paracasei* BGI-N2 (BGI-N2) using integrated genomic and phenotypic analyses. The complete genome of BGI-N2 comprised 3,102,337 bp with a 46.4% GC content and 2,937 coding sequences, including multiple genes related to carbon source fermentation, gastrointestinal tolerance, and antioxidant activities. Furthermore, genomic analysis confirmed the favorable safety profile of BGI-N2. In vitro, this strain showed robust tolerance to extreme gastrointestinal conditions and significant inhibition against common enteropathogens. In constipated zebrafish, it enhanced motility by increasing motilin (MTL) and 5-hydroxytryptamine (5-HT) while decreasing nitric oxide levels. Mechanistically, BGI-N2 upregulated *kitla*, *kitlb*, and *kitb* genes to promote Cajal interstitial cell development, while enhancing 5-HT accumulation through coordinated regulation of biosynthetic genes (*tph1a*, *tph1b*, *tph2*) and the transporter gene (*sert*). This study provides comprehensive genomic insights for BGI-N2 while establishing a theoretical foundation for understanding host-microbe interactions in intestinal motility disorders.

**Supplementary Information:**

The online version contains supplementary material available at 10.1186/s12866-026-04831-0.

## Introduction

Constipation is a functional bowel disorder primarily characterized by reduced frequency and hard, lumpy, or dry stool passage. This condition is frequently accompanied by difficulties during defecation and a sensation of incomplete evacuation. When these symptoms persist for an extended period, a diagnosis of chronic constipation may be established [1]. Epidemiological studies indicate that approximately 15% of the global population is affected by constipation, significantly compromising their quality of life [2, 3]. Current therapeutic strategies are broadly categorized into non-pharmacological and pharmacological interventions. Non-pharmacological approaches include dietary modifications and increased fluid intake, which aim to alleviate symptoms, however, their efficacy is limited by individual variations in intestinal absorption and may not be effective for all patients. Pharmacological treatments encompass oral agents such as polyethylene glycol (PEG) and laxatives, which carry risks of leading to laxative dependence and may cause adverse effects such as nausea and vomiting. Consequently, investigating novel treatment modalities remains imperative.

Research indicates a close association between the development and progression of constipation and gut microbiota dysbiosis. Patients with constipation frequently exhibit a decrease in beneficial bacteria and a potential increase in opportunistic pathogens, such as *Escherichia coli* and *Staphylococcus aureus* [4]. As key modulators of the gut microenvironment, probiotics show promise for improving constipation by regulating the gut microbiota. Specifically, certain probiotics can secrete bacteriocins to selectively inhibit or eliminate harmful bacteria in the gut, thereby creating favorable conditions for the growth of beneficial gut microbiota [5]. Simultaneously, short-chain fatty acids (SCFAs) produced by probiotics can activate the enteric nervous system, thereby promoting colonic peristalsis [6]. Furthermore, studies suggest that some probiotics may alleviate constipation by regulating the expression of neurotransmitters such as 5-hydroxytryptamine (5-HT) [7, 8]. These findings provide a robust scientific basis for using probiotics to modulate the gut microecology and alleviate constipation.

However, probiotics exhibit strain-specific effects, as even different strains within the same species vary significantly in genetic background and probiotic potential. While mammalian experiments and clinical trials represent the gold standard for validating strain functionality, their considerable experimental complexity, prolonged duration, high cost, and limited throughput render them impractical for large-scale preliminary screening of candidate strains [9]. Advanced high-throughput sequencing technologies have made complete genome sequencing a valuable tool for rapidly assessing probiotic candidates. It allows comprehensive analysis of the genetic makeup of probiotic strains, thereby enabling efficient prediction of their probiotic properties [10]. Simultaneously, the zebrafish model has emerged as a powerful system for functional validation owing to its high genetic conservation with humans, optical transparency of larvae facilitating direct observation, short life cycle, low maintenance cost, and compatibility with high-throughput screening [11, 12]. In particular, zebrafish models of constipation can be effectively induced pharmacologically, and techniques such as Nile Red staining allow quantitative evaluation of test compounds for their ability to promote intestinal motility [13, 14]. Thus, an integrated multi-dimensional strategy combining complete genome sequencing, in vitro assays, and in vivo zebrafish models provides a rapid, efficient, and thorough approach for functional analysis of probiotic strains. This methodology holds significant promise for accelerating the development and application of probiotics with targeted potential functions, such as constipation alleviation.

BGI-N2 was isolated from the feces of a healthy Chinese male and initially identified as a promising probiotic candidate. To evaluate its beneficial characteristics and potential applications in digestive health improvement, complete genome sequencing was performed for comprehensive genetic analysis, and a zebrafish model was established to assess the effectiveness in enhancing intestinal motility and alleviating constipation. This study aims to establish a solid theoretical and experimental foundation for the future development of BGI-N2 in constipation management.

## Materials and methods

### Bacterial strains and culture conditions

The strain BGI-N2 was isolated from the feces of a healthy Chinese male and selected from our in-house Cultivated Genome Reference 2 (CGR2) library (original strain name AM33-7BH) [15]. The BGI-N2 strain and the reference strain *Lacticaseibacillus rhamnosus* GG (LGG) were cultured in MRS broth (Hope Bio-Technology, Qingdao, China) at 37 °C under anaerobic conditions (90% N_2_, 5% CO_2_, 5% H_2_) for 48 h. Four pathogenic strains, including *Escherichia coli* ATCC 25,922, *Staphylococcus aureus* ATCC 29,213, *Enterobacter cloacae* ATCC 700,258, and *Pseudomonas aeruginosa* ATCC 27,853, were cultured in BHI medium (Aobox Biotechnology, Beijing, China) at 37 °C for 24 h.

### Complete genome sequencing, assembling, and annotation

Genomic DNA of BGI-N2 was extracted using the Magen MagPure DNA Kit (MagenBiotech, Guangzhou, China). Library preparation was performed according to the protocol described by Liang et al. [16]. For sequencing, short-read data were generated using the DNBSEQ-T5 platform (MGI, Shenzhen, China), and long-read data using the CycloneSEQ-WT02 platform (MGI, Shenzhen, China). A hybrid assembly of short-read and long-read data was generated with Unicycler v0.4.8 to obtain the complete genome sequence [17]. The genome assembly quality was assessed using CheckM v1.0.2 [18], and genome annotation was performed with Prokka v1.14.6 [19]. Furthermore, the Proksee server (https://proksee.ca/) was used for genome visualization analysis [20]. Subsequently, complete genome sequences of all *L. paracasei* strains available from NCBI were clustered based on an Average Nucleotide Identity (ANI) threshold greater than 99%. From each cluster, one strain demonstrating the highest genome completeness and lowest level of contamination was selected for subsequent ANI calculation and phylogenetic tree construction. The representative strain BGI-N2 was also included in these analyses. Species delineation was validated with GTDB-Tk v2.4.1, and ANI values were computed using fastANI v1.34 to systematically determine the phylogenetic position of strain BGI-N2 [21, 22]. Functional annotation was conducted through the EggNOG Database [23] (http://eggnog5.embl.de/), resulting in three types of annotations: Clusters of Orthologous Groups (COG) for functional classification, Kyoto Encyclopedia of Genes and Genomes (KEGG) for metabolic pathway analysis, and Carbohydrate-Active Enzymes (CAZymes) for carbohydrate-active enzyme identification. Additionally, secondary metabolite biosynthetic gene clusters were predicted by AntiSMASH bacterial version (https://antismash.secondarymetabolites.org) [24].

### Genomic safety assessment

The genomic safety of BGI-N2 was assessed by screening its genome for plasmids, antibiotic resistance genes (ARGs) and virulence factors (VFs). Plasmid sequences were predicted using the geNomad v1.11.0 and PlasmidFinder (https://cge.food.dtu.dk/services/PlasmidFinder/) tools [25, 26]. ARGs were predicted using the Resistance Gene Identifier (RGI) tool from the Comprehensive Antibiotic Resistance Database (CARD) (https://card.mcmaster.ca/) under default parameters [27]. Subsequently, the protein sequences were aligned against the core dataset (SetA) of the Virulence Factor Database (VFDB) (http://www.mgc.ac.cn/VFs/) using BLASTP. Hits with sequence identity > 60% and coverage > 50% were retained as reliable VFs [28].

### Digestive tract environment tolerance test

The activated BGI-N2 strains were cultured in MRS broth at 37 °C for 24 h, after which the cells were harvested by centrifugation (6,000 rpm, 10 min) and washed twice with sterilized PBS (pH 7.4). The cell pellets were resuspended in 10 mL of simulated gastric fluid, simulated intestinal fluid, or MRS broth supplemented with 0.3% bile salts as described by Wang et al. [29]. The resuspended cultures were incubated anaerobically at 37 °C for 2 h. Viable bacterial counts were determined at 0 and 2 h using the plate counting method to assess the tolerance of BGI-N2 to the human digestive tract environment.

### Antibacterial activity test

The antibacterial activity of BGI-N2 strain cell-free supernatant (CFS) against pathogens (*E. coli* ATCC 25922, *S. aureus* ATCC 29213, *E. cloacae* ATCC 700258, and *P. aeruginosa* ATCC 27853) was assessed according to the method described by Wang et al. [29]. Briefly, BGI-N2 was cultured, centrifuged (7,000 rpm, 5 min), and supernatant filtered (0.22 μm) for testing. 100 µL of 2×BHI broth was dispensed into each well and mixed with an equal volume of CFS, sterile water (blank control), or 50 µg/mL antibiotic (positive control). Then all wells were inoculated with 50 µL pathogen suspension and cultivated at 37 °C for 24 h. Each treatment was performed in triplicate. OD_600_ was measured at 0 h and 24 h to quantify bacterial density, and the inhibition rate was calculated.

### Zebrafish experiment

#### Zebrafish culture

Wild-type zebrafish (AB strain) were obtained from Hangzhou Hunter Biotechnology Co., Ltd., and maintained at 28 °C in aquaculture water. Embryos were collected through natural pairwise mating, and 5 days post-fertilization (5 dpf) larvae were randomly distributed into 6-well plates (30 larvae/well) for experiments. At the endpoint, larvae were euthanized using a hypothermic shock method, which involved rapid immersion in an ice-water mixture (0–4 °C) for ≥ 30 min to minimize distress. Death was confirmed by the irreversible cessation of opercular movement and heartbeat for over 60 s. All animal experiments were conducted in agreement with the Guide for the Care and Use of Laboratory Animals and approved by the Institutional Review Board of BGI (Approval No. BGI-IRB A24032).

#### Maximum tolerable concentration assay

Zebrafish were randomly distributed into 6-well plates and exposed to BGI-N2 suspensions at concentrations ranging from 3.294 × 10^7^ to 5.270 × 10^8^ CFU/mL for 24 h, while the normal control (NC) and model control (MOD) groups did not receive BGI-N2 treatment. After 24 h incubation, probiotics were removed, and all groups were stained with 50 ng/mL Nile Red for 3 h to label intestinal contents. Subsequently, constipation was induced in experimental groups (excluding the NC group) by exposure to 1 µg/mL aluminum sulfate (Shanghai Yien Chemical Technology Co., Ltd., Shanghai, China) for 6 h. Survival rates were recorded to determine the maximum tolerable concentration (MTC).

#### Intestinal retention assay

The intestinal retention capacity of BGI-N2 was evaluated in zebrafish using the CM-Dil fluorescence labeling method adapted from Chen et al. [30]. BGI-N2 and the reference strain LGG were labeled with 10 µg/mL CM-Dil at 37 °C for 20 min. Zebrafish larvae were randomly distributed into 6-well plates and exposed to probiotic suspensions (1.0 × 10^8^ CFU/mL) in aqueous solution for 24 h. After removing probiotics, larvae were maintained and sampled at 0, 6, and 24 h post-exposure. For each time point, ten zebrafish were randomly imaged under fluorescence microscopy using NIS-Elements D 3.20 software to quantify intestinal fluorescence intensity.

#### Assessment of intestinal transit capacity

Three doses of BGI-N2 were selected for the constipation study based on MTC results and supported by previous studies that explored effective probiotic doses in zebrafish models [31, 32], including a low dose (LD, 5.0 × 10^7^ CFU/mL), a medium dose (MD, 1.0 × 10^8^ CFU/mL), and a high dose (HD, 2.0 × 10^8^ CFU/mL). The control groups consisted of a normal control (NC, untreated), a model control (MOD, constipation-induced only), and a positive control (PC, administered with 50.0 µg/mL domperidone). The intervention followed the same protocol as utilized in the MTC assay. Following treatment, ten zebrafish from each group were randomly selected and subjected to fluorescence microscopy imaging with NIS-Elements D 3.20 software to quantify intestinal fluorescence intensity.

#### Assessment of intestinal nitric oxide concentration

Following the intestinal transit capacity assay protocol, the nitric oxide (NO) sensitive fluorescent probe DAF-FM DA (Yeasen Biotechnology Co., Ltd., Shanghai, China) was applied at a concentration of 5 µmol/L for 1 h to replace Nile Red for assessing the effect of BGI-N2 on intestinal NO concentration in zebrafish.

#### 5-HT and MTL assay

5-HT and MTL levels were measured using specific ELISA kits (Shanghai Enzyme-Linked Biotechnology Co., Ltd., Shanghai, China) according to the instructions.

#### Quantitative real-time PCR analysis

Samples from each group were selected for RNA extraction using the Universal RNA Extraction Kit (Foshan Aowei Biotechnology Co., Ltd., Foshan, China). Reverse transcription and quantitative real-time PCR (qPCR) were performed with the FastKing One Step RT-qPCR Kit (Tiangen Biotech Co., Ltd., Beijing, China). The targeted genes included *sert*, *kitla*, *kitb*, *kitlb*, *htr1aa*, *tph1a*, *tph1b*, and *tph2*, with primer sequences provided in Table S1. Experimental procedures and conditions followed previously published methods [31]. Relative gene expression was calculated using the 2^–ΔΔCt^ method, with *β-actin* as an internal control.

### Statistical analysis

The data were analyzed using SPSS 27.0. Statistical analyses included t-tests and one-way analysis of variance (ANOVA). Results were expressed as mean ± standard deviation (Mean ± SD). *p* < 0.05 was considered statistically significant. All graphs were created using GraphPad Prism 9.5 (San Diego, CA, USA).

## Results

### General genomic feature of BGI-N2

**Fig. 1:**
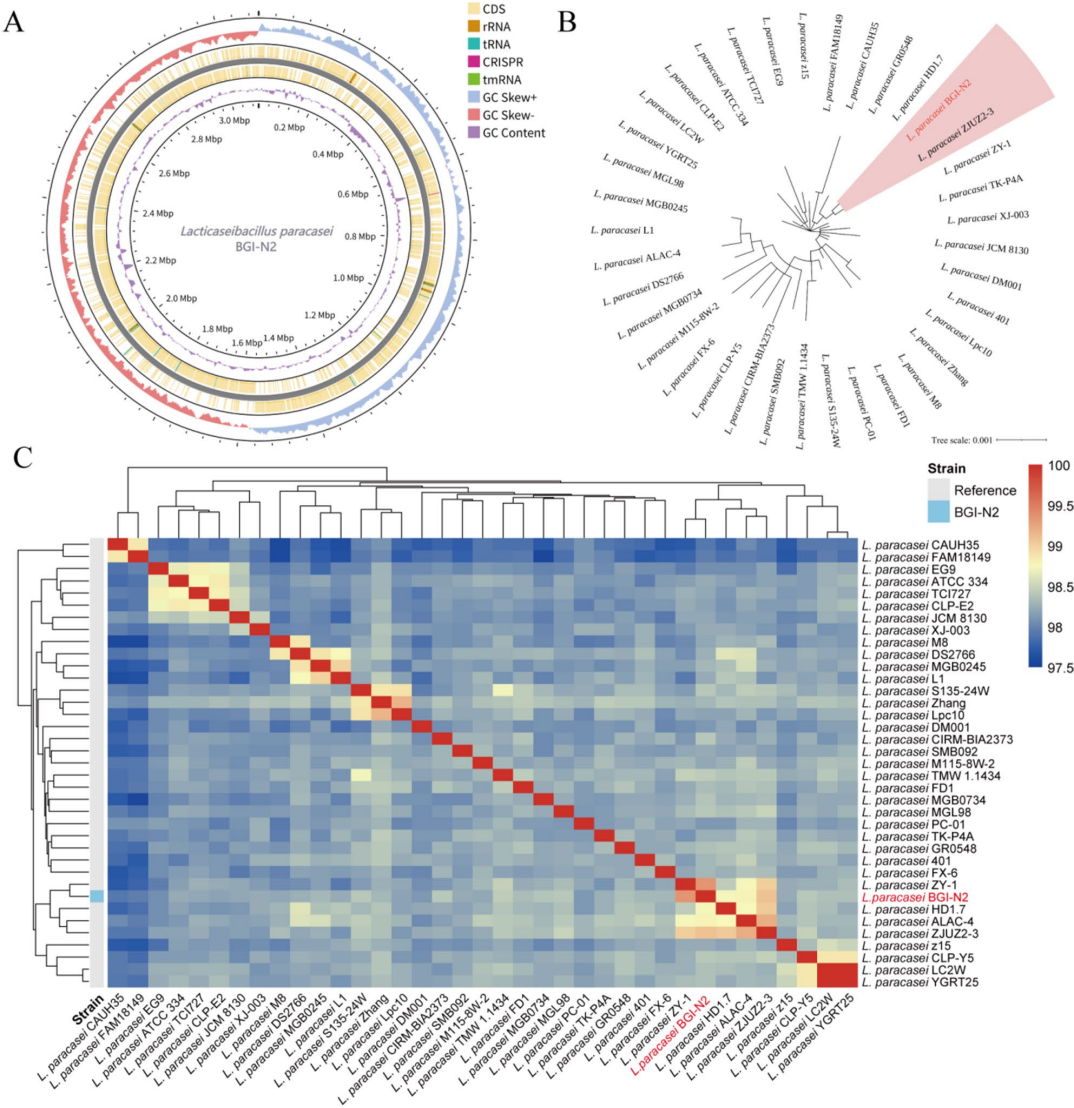
General genomic feature of BGI-N2. **A** The genome circle map of BGI-N2. **B** Evolutionary placement of BGI-N2 reconstructed from core-genome alignment. **C** Heatmap of ANI values between BGI-N2 and other *L. paracasei* strains

The genome circle map of BGI-N2 is displayed in Fig. 1A. It consisted of a single contig spanning 3,102,337 base pairs (bp), with an average GC content of 46.4%. Prokka analysis predicted that the genome contained 2,937 coding sequences (CDS), 59 tRNA genes, 15 rRNA genes, and 1 tmRNA gene. According to the ANI clustering results, all *L. paracasei* strains were classified into 37 clusters. Phylogenetic analysis of these strains revealed that BGI-N2 and *L. paracasei* ZJUZ2-3 clustered within the same evolutionary branch (Fig. 1B). Furthermore, ANI analysis indicated that BGI-N2 exhibited ANI values greater than 99% with both *L. paracasei* ZY-1 and *L. paracasei* ZJUZ2-3, suggesting a high degree of genomic homology (Fig. 1C).

### Functional annotation of BGI-N2

**Fig. 2:**
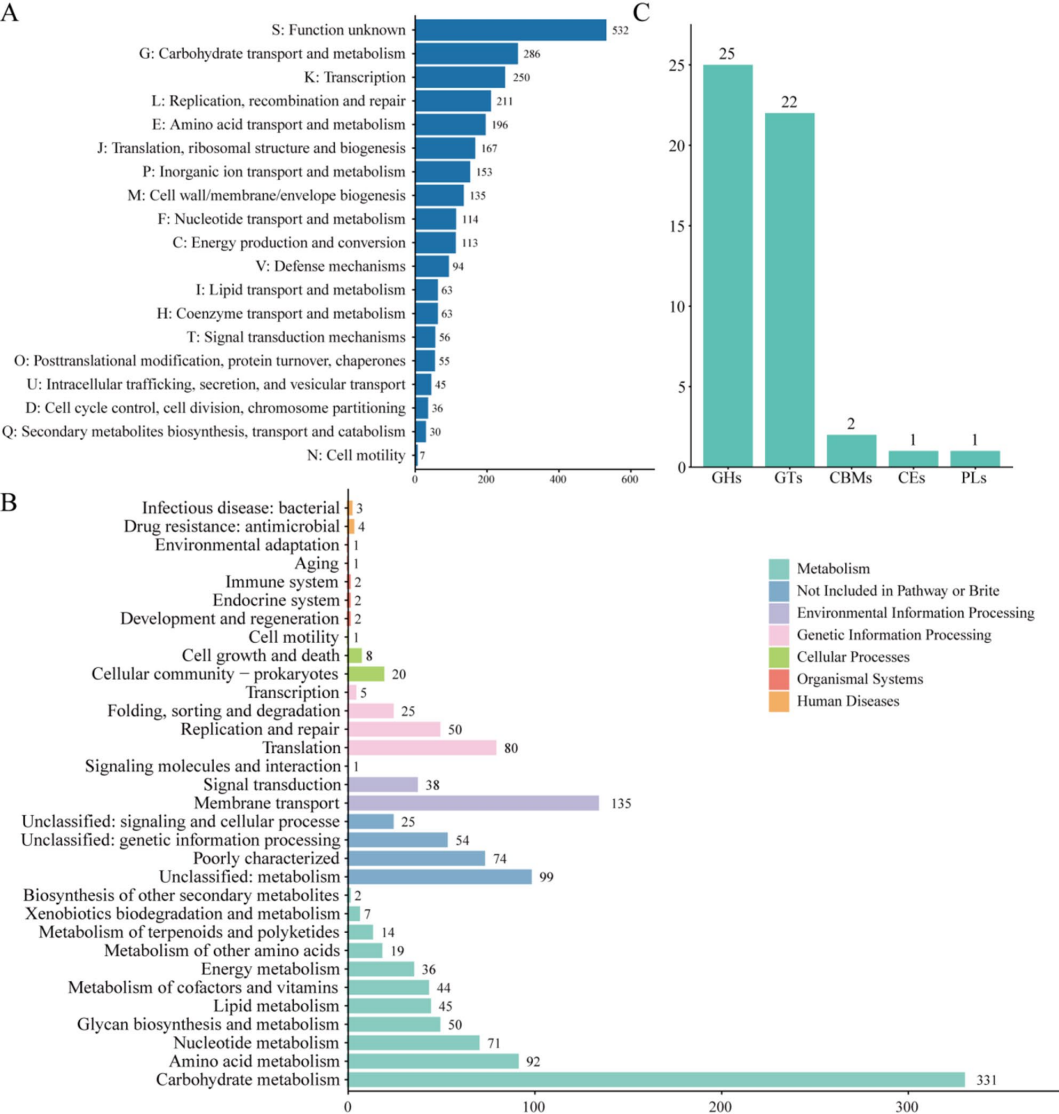
Functional annotation of BGI-N2. Represent functional annotation distributions of the BGI-N2 genome based on (**A**) COG, (**B**) KEGG, and (**C**) CAZymes databases

Functional classification of 2,752 genes was performed with the EggNOG database, revealing that most were associated with cellular metabolism and signal transduction (Fig. 2A). The five most abundant COG classes were G (Carbohydrate transport and metabolism, 286 genes), K (Transcription, 250 genes), L (Replication, recombination, and repair, 211 genes), E (Amino acid transport and metabolism, 196 genes), and J (Translation, ribosomal structure, and biogenesis, 167 genes). Further genomic interrogation of BGI-N2 identified antioxidant-related genes (*mntH*, *msrA*/*B*, *ndh*, *nox*, *npr*, *nrdH*, *poxL*, *tpx*, *trxA*/*B*, and *ahpC*) (Table S2). The presence of these conserved functional modules substantiates the underlying probiotic properties of BGI-N2. In addition, KEGG pathway analysis annotated 1341 KEGG Orthologs, most of which were associated with metabolism, genetic information processing, and environmental information processing (Fig. 2B). Regarding carbohydrate metabolism, the genome of BGI-N2 contains a total of 51 genes encoding CAZymes, including 25 genes encoding glycoside hydrolases (GHs) and 22 genes encoding glycosyl transferases (GTs) (Fig. 2C). These CAZymes may play crucial roles in glycosidic bond degradation, modification, and synthesis.

### Genomic safety assessment of BGI-N2

Integrated analysis revealed the absence of plasmids in BGI-N2. Genomic analysis against the CARD database identified a putative small multidrug efflux pump gene associated with *qacJ* in BGI-N2 under strict criteria, which potentially confers resistance to disinfecting agents and antiseptics (Table S3). Although sequence similarity was relatively low (38.24%), the high coverage (99.07%) suggested this may represent a novel homolog requiring further functional validation. 12 genes in BGI-N2 showed significant matches to the VFDB, with functions primarily involving adherence, stress survival, immune modulation, and regulation (Table S4). Most of these genes are commonly detected in *L. paracasei* strains, including those encoding Lap, EF-Tu, GroEL, and ClpP proteins [33], indicating that they may represent the conserved intrinsic genes of this species. Notably, the *lap* gene has been experimentally demonstrated to enhance intestinal colonization and reduce pathogen adhesion, contributing to probiotic functions [34]. All the aforementioned genes are chromosomally encoded, which indicates a relatively low potential risk of horizontal gene transfer. Nevertheless, it is necessary to conduct further functional and safety validation for these genes in BGI-N2.

### Gastrointestinal tolerance of BGI-N2

The viability of probiotics under extreme digestive stresses dictates their capacity for intestinal colonization and exertion of health-promoting effects [35, 36]. Genomic annotation revealed that 11 genes associated with acid resistance, 23 with bile salt resistance, and 8 with lysozyme resistance were identified in BGI-N2 (Table 1). Further analysis indicated that BGI-N2 possesses genes encoding the K^+^/H^+^ antiporter (*nhaK*) and F_0_F_1_-ATP synthase subunits (*atpA*~*atpH*), which are likely involved in maintaining proton homeostasis and enhancing acid tolerance. Additionally, the genome contained the *opp* operon (*oppA*~*oppD*, *oppF*), cyclopropane fatty acid synthase (*cfa*), and UDP-galactose mutase (*glf*) genes, which may collectively participate in regulating membrane stability, bile salt efflux, and other processes, thereby enhancing its tolerance to the bile salt environment. Furthermore, genes such as *otaA*, *mprF*, the *dlt* operon (*dltA*~*dltD*), and *gpsA*/*B* were identified, suggesting a role in modifying cell wall and membrane components to confer lysozyme resistance. These functional gene sets constituted a comprehensive genetic arsenal that enabled BGI-N2 to survive under extreme environmental conditions. In vitro assessments demonstrated that BGI-N2 exhibited a high survival rate exceeding 90% in artificial gastric fluid (pH 3.0), artificial intestinal fluid, and 0.3% bile salt solution (Table 2). These findings indicated that BGI-N2 could effectively tolerate the gastrointestinal environment, supporting its potential application as a probiotic.

**Table 1:** Acid resistance, bile salt resistance, and lysozyme resistance genes of BGI-N2

Type	Preferred Name	EC	KEGG	PFAMs
Acid resistance	*nhaK*	-	K03316	Na_H_Exchanger
	*atpB*	-	K02108	ATP-synt_A
	*atpE*	-	K02110	ATP-synt_C
	*atpF*	-	K02109	ATP-synt_B
	*atpH*	-	K02113	OSCP
	*atpA*	3.6.3.14	K02111	ATP-synt_ab ATP-synt_ab_C ATP-synt_ab_N
	*atpG*	-	K02115	ATP-synt
	*atpD*	3.6.3.14	K02112	ATP-synt_ab ATP-synt_ab_N
	*atpC*	-	K02114	ATP-synt_DE ATP-synt_DE_N
	*pyk*	2.7.1.40	K00873	PK PK_C PEP-utilizers
	*plsC*	2.3.1.51	K00655	Acyltransferase
Bile salt resistance	*oppA*	-	K02035 K15580	SBP_bac_5
	*rpsD*	-	K02986	S4 Ribosomal_S4
	*rpsT*	-	K02968	Ribosomal_S20p
	*rpsO*	-	K02956	Ribosomal_S15
	*rpmF*	-	K02911	Ribosomal_L32p
	*rpsA*	-	K02945	S1
	*ppaC*	3.6.1.1	K15986	DHH DHHA2
	*rpsU*	-	K02970	Ribosomal_S21
	*rpsB*	-	K02967	Ribosomal_S2
	*rplS*	-	K02884	Ribosomal_L19
	*rpsP*	-	K02959	Ribosomal_S16
	*rpmB*	-	K02902	Ribosomal_L28
	*rpmA*	-	K02899	Ribosomal_L27
	*rplU*	-	K02888	Ribosomal_L21p
	*glnA*	6.3.1.2	K01915	Gln-synt_C Gln-synt_N
	*rplT*	-	K02887	Ribosomal_L20
	*rpmI*	-	K02916	Ribosomal_L35p
	*glf*	5.4.99.9	K01854	GLF NAD_binding_8
	*oppF*	-	K10823	ABC_tran oligo_HPY
	*oppD*	-	K02031 K15583	ABC_tran oligo_HPY
	*oppC*	-	K15582	BPD_transp_1 OppC_N
	*oppB*	-	K15581	BPD_transp_1
	*cfa*	2.1.1.79	K00574	CMAS
Lysozyme resistance	*oatA*	-	-	Acyl_transf_3
	*mprF*	2.3.2.3	K14205	DUF2156 LPG_synthase_TM
	*dltA*	6.1.1.13	K03367	AMP-binding AMP-binding_C
	*dltB*	-	K03739	MBOAT
	*dltC*	6.1.1.13	K14188	PP-binding
	*dltD*	-	K03740	DltD
	*gpsA*	1.1.1.94	K00057	NAD_Gly3P_dh_C NAD_Gly3P_dh_N
	*gpsB*	-	-	DivIVA

**Table 2:** Survival rates of BGI-N2 in simulated Gastrointestinal digestive fluids

Digestive Tract Environment	Survival Rate (%)
Simulated gastric fluid (pH 3.0)	97.80 ± 1.32
Simulated intestinal fluid	99.50 ± 0.46
0.3% bile salt solution	90.40 ± 1.98

### Antimicrobial activity of BGI-N2

**Fig. 3:**
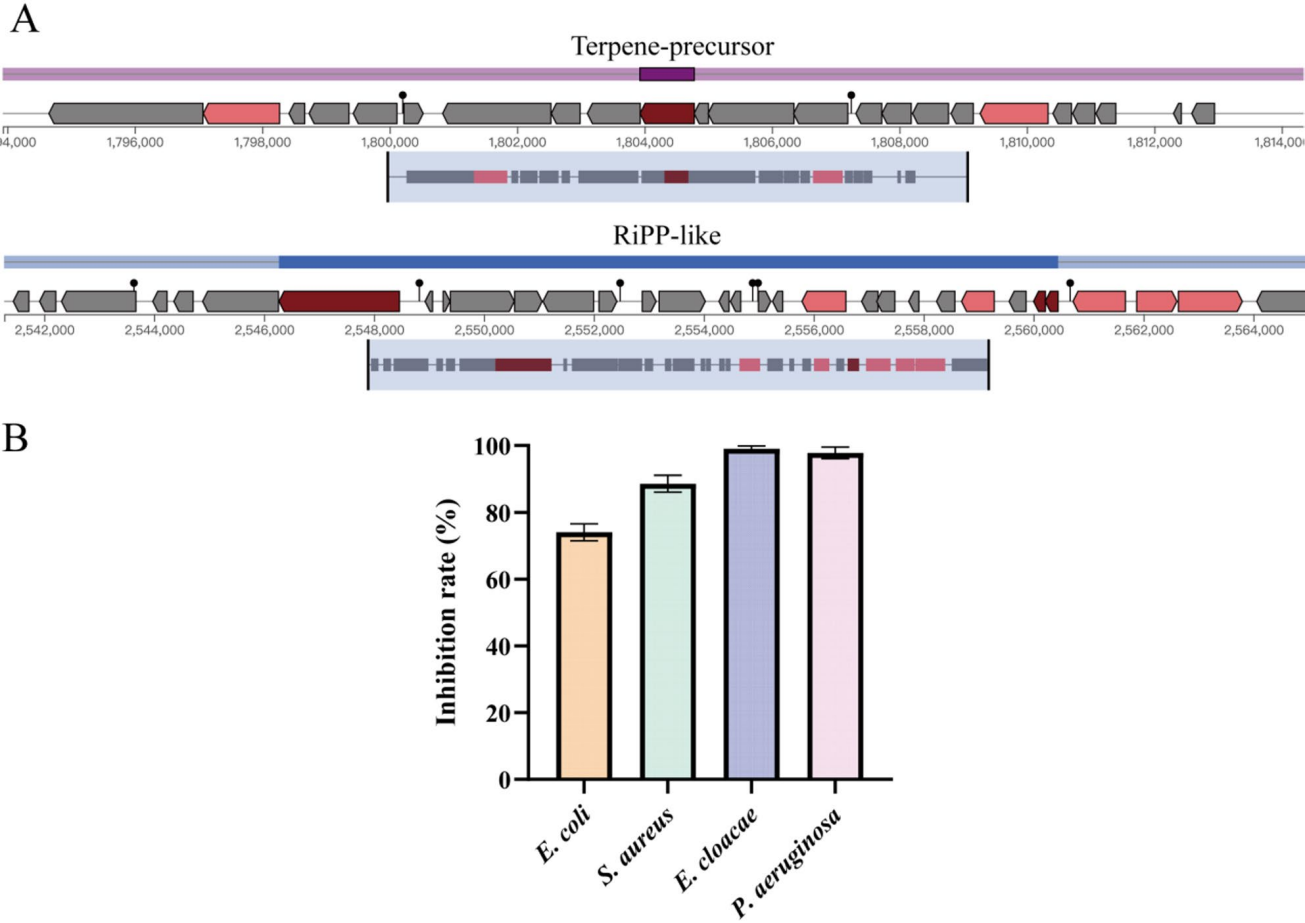
Antimicrobial activity of BGI-N2. **A** Two secondary metabolite gene clusters in the BGI-N2 genome identified via AntiSMASH annotation. **B** The inhibition rates of BGI-N2 on the growth of pathogens

Inhibiting pathogen growth is a key mechanism through which probiotics benefit the host by modulating the gut microbiota. Genomic analysis of BGI-N2 revealed genes associated with the synthesis of antimicrobial substances, including *alsS* (diacetyl), *nox* (hydrogen peroxide), and *ldh* (lactate) (Table S2), suggesting a genetic capacity for pathogen inhibition. Furthermore, AntiSMASH annotation identified two secondary metabolite gene clusters, a terpene-precursor cluster and a RiPP-like (ribosomally synthesized and post-translationally modified peptide) cluster that harbors the bacteriocin-synthesis gene *lcnD* (Fig. 3A). Supporting this genetic potential, in vitro antibacterial assays demonstrated that BGI-N2 exhibited significant inhibitory activity against *E. coli* ATCC 25,922, *S. aureus* ATCC 29,213, *E. cloacae* ATCC 700,258, and *P. aeruginosa* ATCC 27,853 with inhibition rates of 74.10 ± 2.53%, 88.60 ± 2.49%, 97.80 ± 1.74%, and 99.00 ± 0.89%, respectively. (Fig. 3B).

### Intestinal retention of BGI-N2

**Fig. 4:**
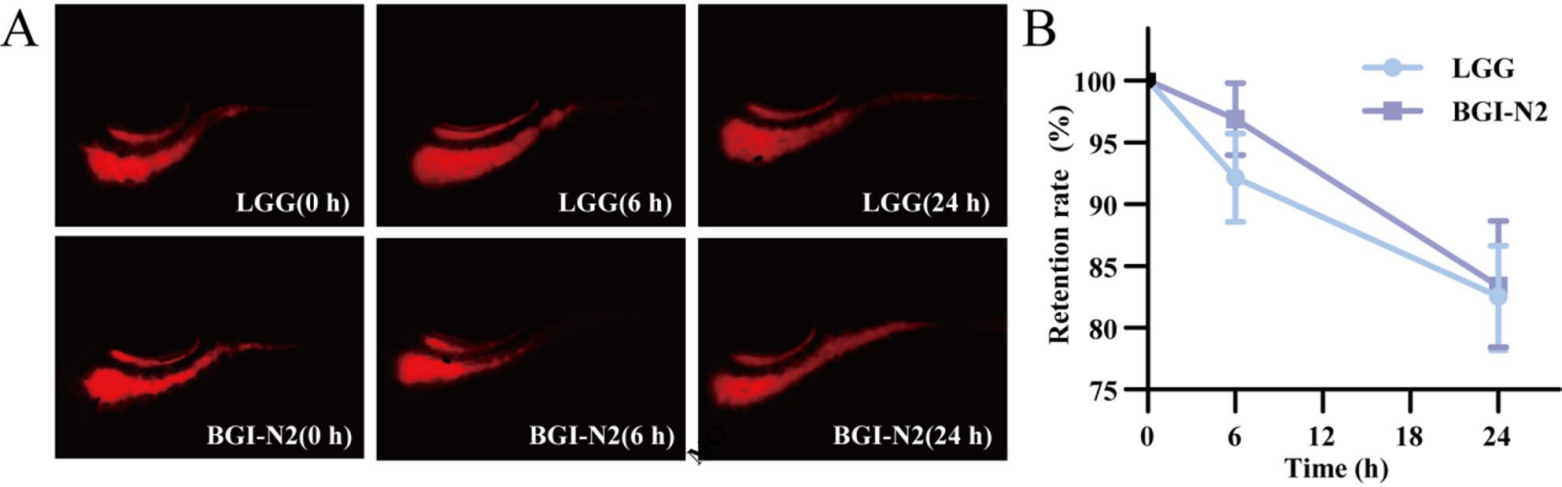
The retention abilities of BGI-N2 and LGG in the zebrafish intestine. **A** Representative fluorescence micrographs of zebrafish intestines after administration with CM-Dil-labeled BGI-N2 and LGG. **B** Retention rates of BGI-N2 and LGG in zebrafish intestines at 6 h and 24 h

Adhesion capability, a key determinant for probiotic persistence in the intestinal tract, extends their beneficial effects [37, 38]. Among the seven adhesion-related genes identified in BGI-N2 (Table S2), *mapA*, *lspA*, *eno*, and *tuf* may contribute to adhesion by specifically recognizing and binding to mucins, acting as structural adhesins that directly bind to host cell surface receptors, and functioning as surface enzymes that interact with the extracellular matrix, respectively. Furthermore, seven genes supporting intestinal adaptation likely enhance the colonization of BGI-N2 through multiple functions, encompassing capsular polysaccharide synthesis (e.g., *cps2E*, *cps2D*), stress response (*rpoN*, *rpoE*), and quorum sensing (*luxS*) (Table S2). The synergistic effects of these genes underpin the intestinal colonization and probiotic functions of BGI-N2. To validate its colonization capacity, the intestinal retention of BGI-N2 was compared with that of the commercial reference strain *L. rhamnosus* LGG in a zebrafish model (Fig. 4A). As shown in Fig. 4B, twenty-four hours after administration, BGI-N2 demonstrated a retention rate of 83.37 ± 5.26%, with no significant difference observed compared to LGG. This robust colonizing ability provided a critical foundation for its potential to exert more sustained and stable probiotic effects.

### Effect of BGI-N2 on constipation relief

**Fig. 5:**
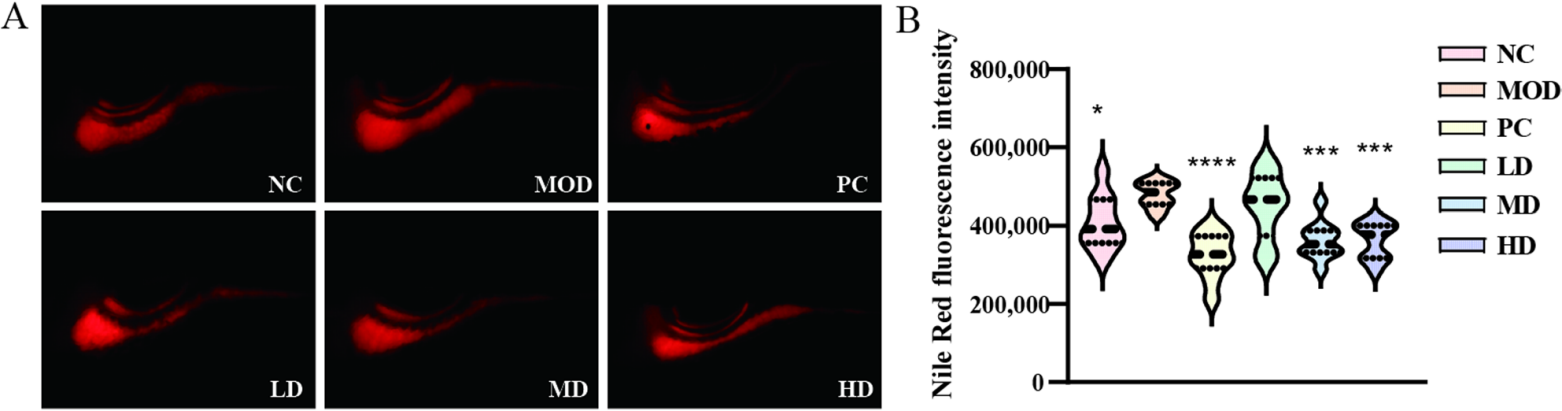
The intestinal motility and constipation relief of BGI-N2 in the zebrafish intestine. **A** Representative fluorescence micrographs of Nile Red-stained intestinal contents in zebrafish. **B** Quantitative analysis of Nile Red in fluorescence intensity. * indicates statistically significant differences compared to the MOD group (* *p* < 0.05, *** *p* < 0.001, **** *p* < 0.0001)

To evaluate the safety of BGI-N2 in zebrafish, different concentrations of BGI-N2 suspensions were added to the aquarium water. All zebrafish survived after exposure to BGI-N2 suspensions (Table S5), indicating no acute toxicity within the tested concentration range. The absence of mortality confirmed its suitability for subsequent probiotic efficacy studies. Additionally, Nile Red was used as a fluorescent tracer for intestinal contents to evaluate the impact of BGI-N2 on intestinal motility and emptying capacity (Fig. 5A). Results demonstrated that the intestinal contents in the MOD group were significantly greater than those in the NC group (*p* < 0.05), confirming successful establishment of the constipation model. Following exposure to BGI-N2, the MD and HD groups exhibited significantly reduced intestinal contents compared to the MOD group (*p* < 0.001) (Fig. 5B). These findings indicate that medium and high doses of BGI-N2 significantly enhanced intestinal motility and emptying capacity in zebrafish.

### Effect of BGI-N2 on intestinal NO concentration

**Fig. 6:**
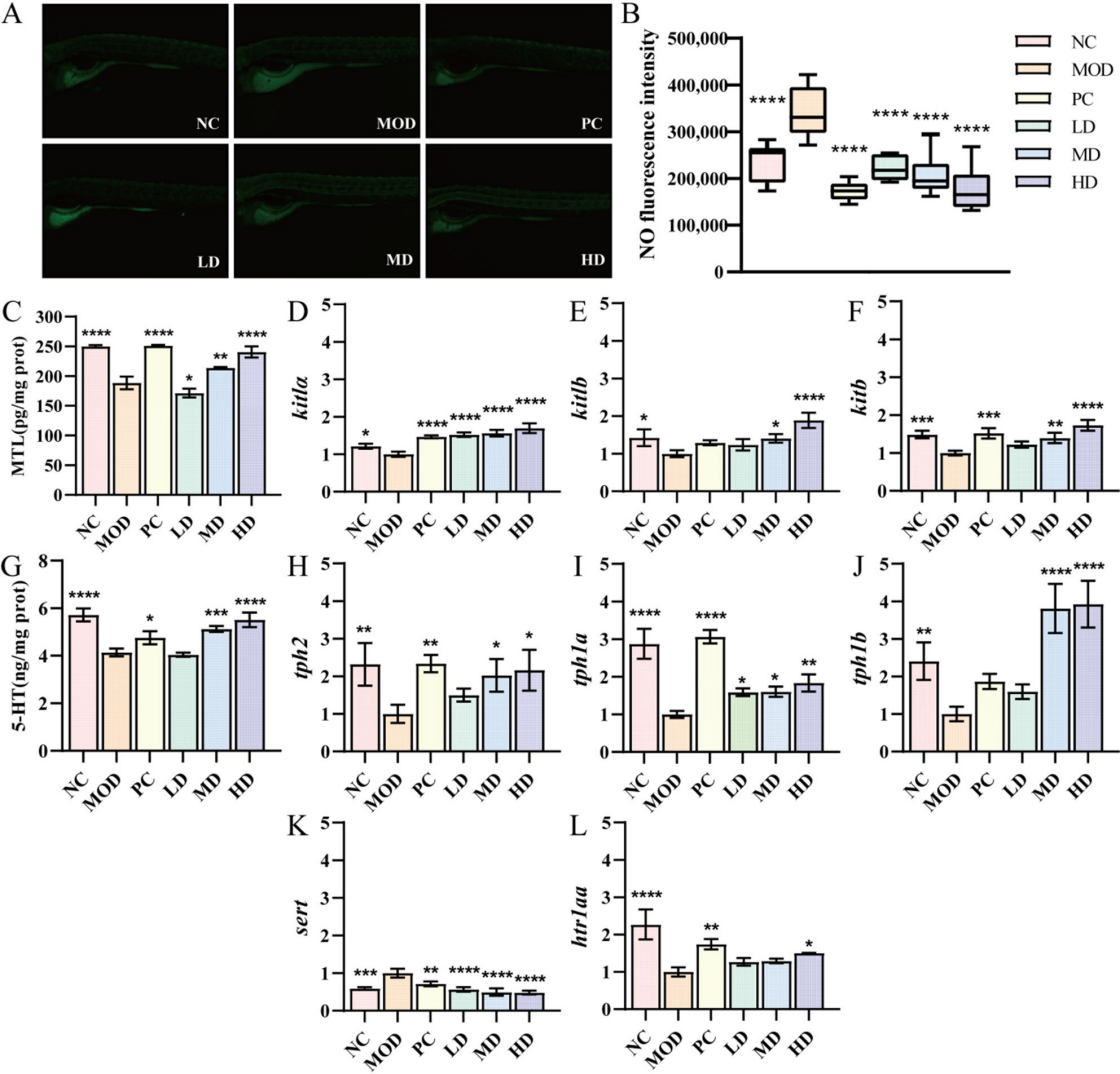
Effects of BGI-N2 on NO, MTL, and 5-HT levels and related gene expression in zebrafish. **A** Representative fluorescence micrographs of intestinal NO levels in zebrafish. B Quantitative analysis of NO fluorescence intensity. **C** MTL secretion levels under different treatments. **D**-**F** Expression of *kitla*, *kitlb*, and *kitb* genes across all groups. **G** 5-HT secretion levels under different treatments. **H**-**L** Expression of *tph2*, *tph1a*, *tph1b*, *sert*, and *htr1aa* genes across different groups. * indicates statistically significant differences compared to the MOD group (* *p* < 0.05, ** *p* < 0.01, *** *p* < 0.001, **** *p* < 0.0001)

NO, as an inhibitory neurotransmitter released by enteric inhibitory neurons, can induce intestinal dysmotility when excessively synthesized [39, 40]. NO levels in zebrafish intestines were quantified using an NO-sensitive fluorescent probe (Fig. 6A). The MOD group exhibited the highest NO concentration. Following BGI-N2 intervention, intestinal NO decreased significantly (*p* < 0.0001), with a dose-dependent reduction observed across increasing bacterial concentrations (Fig. 6B). This decline facilitates restoration of intestinal motor function, suggesting that the alleviation of constipation symptoms by BGI-N2 may be mediated through the downregulation of intestinal NO.

### Effects of BGI-N2 on MTL secretion and kit pathway gene expression

Research demonstrated that treatment with BGI-N2 effectively elevated MTL levels in zebrafish. Specifically, the HD group exhibited significantly higher MTL secretion than the MOD group (*p* < 0.0001), achieving concentrations comparable to the NC and PC groups (Fig. 6C). Furthermore, BGI-N2 intervention markedly modulated interstitial cells of Cajal (ICC) pacemakers and excitatory conductors for intestinal motility that regulate smooth muscle contraction. The Kit signaling pathway (*kitla*, *kitlb*, and *kitb*) is pivotal in ICC development, maintenance, and functionality [41, 42]. qPCR analysis demonstrated dose-dependent upregulation of *kitla*, *kitlb*, and *kitb* gene expression in BGI-N2-treated zebrafish, with HD group expression exceeding the PC group (Fig. 6D-F). These findings indicated that BGI-N2 enhanced intestinal motility by concurrently stimulating MTL secretion and upregulating key Kit pathway components.

### Effects of BGI-N2 on 5-HT accumulation

5-HT is critical in alleviating constipation by regulating intestinal motility and secretion [43]. In this study, BGI-N2 significantly increased 5-HT levels in zebrafish at both MD and HD groups (Fig. 6G) (MD *p* < 0.001, HD *p* < 0.0001). qPCR analysis further revealed upregulation of 5-HT biosynthetic pathway genes, *tph2* (*p* < 0.05), *tph1a* (MD *p* < 0.05, HD *p* < 0.01), and *tph1b* (*p* < 0.0001) in MD and HD groups (Fig. 6H-J). Notably, the expression of *tph1b* (encoding tryptophan hydroxylase) in the MD and HD groups was more than double that in the PC group. Conversely, the 5-HT transporter gene *sert* exhibited significantly reduced expression compared to the MOD group (*p* < 0.0001), with a decrease of approximately 33% in the HD group (Fig. 6K). Additionally, the expression of the 5-HT receptor gene *htr1aa* was significantly increased in the HD group (Fig. 6L) (*p* < 0.05). These results demonstrated that BGI-N2 enhanced 5-HT accumulation by simultaneously promoting biosynthesis and inhibiting transport, thereby alleviating constipation symptoms.

## Discussion

In recent years, the potential of probiotics in managing constipation has gained increasing attention. Genomic analysis revealed that strain BGI-N2 possesses a favorable safety profile, and carries multiple genes associated with probiotic functions. Notably, BGI-N2 exhibits distinct features compared to reported *L. paracasei* strains, it harbors rare antimicrobial genes (*alsS* and *lcnD*), and demonstrates exceptional tolerance to low-pH gastric stress [44]. In vitro assays further demonstrated robust gastrointestinal tolerance and notable antibacterial activity of the strain. In vivo investigations confirmed that BGI-N2 significantly alleviated constipation symptoms by reducing NO levels while increasing 5-HT and MTL concentrations in zebrafish.

Safety and effective intestinal colonization are fundamental prerequisites for probiotics to exert beneficial functions and enable further applications. Genomic analysis indicated that BGI-N2 exhibits a favorable safety profile and harbors multiple functional genes that support its survival and colonization. Specifically, the genes *atpA*/*B*, *oppA*, and *otaA* enhance the strain’s resistance to gastrointestinal conditions, bile salts, and lysozyme, respectively [45–48]. Meanwhile, *ldh*, *nox*, and *alsS* are involved in synthesizing lactate, hydrogen peroxide, and diacetyl. These metabolites have been shown to inhibit the growth of various enteropathogens [49, 50]. Consistent with these genetic features, BGI-N2 demonstrated a high survival rate in simulated gastrointestinal harsh environments, and showed effective colonization in zebrafish. Furthermore, in vitro antibacterial assays confirmed that BGI-N2 effectively inhibit the growth of multiple enteropathogens. These findings suggested that BGI-N2 not only directly supplements beneficial bacteria but may also improve intestinal function by inhibiting pathogen proliferation and optimizing the gut microbial balance. Notably, several studies have demonstrated that probiotics alleviate constipation by reducing the relative abundance of intestinal pathogens [51–53], indicating that BGI-N2 may exert its probiotic effects through a similar mechanism. In summary, integrated evidence from genomic analysis and in vitro experiments indicated that BGI-N2 possesses the potential to survive and exert beneficial functions in the complex intestinal environment, thereby laying a solid foundation for alleviating constipation.

Zebrafish possesses characteristics such as high fecundity, early optical transparency, and relatively conserved gut functions with mammals, making it a valid model for studying on host health [54]. In this study, BGI-N2 alleviated aluminum sulfate-induced constipation in zebrafish through multiple mechanisms. Specifically, BGI-N2 downregulated NO levels, which may relieve the inhibition of intestinal motility and thereby promote intestinal transit, consistenting with observations from similar studies by Liang et al. [55]. Furthermore, MTL is regarded as a classic prokinetic hormone, and its decreased levels diminish the contractile stimulation on intestinal smooth muscle, thereby resulting in insufficient intestinal motility [56]. Our results demonstrated that BGI-N2 intervention significantly elevated MTL levels in constipated zebrafish. Additionally, ICC act as intestinal pacemakers and play a key role in maintaining rhythmic intestinal motility [57]. Abnormally elevated NO can impair ICC quantity and function, delaying intestinal transit [40]. Correspondingly, zebrafish lacking the *kita* gene (a key receptor for ICC) exhibit significantly reduced intestinal contraction frequency [58]. Previous studies have shown that probiotic intervention can ameliorate intestinal motility disorders by reducing NO and enhancing *Kit* expression [59]. Consistent with these findings, we observed similar regulatory effects in our study. Therefore, we speculate that enhancing the functional integrity or activity of the ICC via this pathway, thereby restoring spontaneous intestinal peristalsis, may be a key mechanism by which BGI-N2 alleviates constipation.

5-HT as a core neurotransmitter regulating intestinal peristalsis and sensory function, it triggers peristaltic reflexes by acting on intestinal neurons and sensory nerve endings [31]. In this study, BGI-N2 intervention significantly increased intestinal 5-HT levels in zebrafish and enhanced 5-HT signaling through multi-level genetic regulation. qPCR results showed that BGI-N2 significantly upregulated the expression of *tph1a*, *tph1b*, and *tph2* genes, which encode rate-limiting enzymes in 5-HT synthesis. The underlying mechanism of this process may be closely associated with probiotic metabolic activity. For instance, SCFAs produced by probiotics, particularly butyrate, have been confirmed to upregulate the transcriptional expression of *tph1* [60–62]. Meanwhile, the upregulated expression of the 5-HT receptor gene *htr1aa* enhanced the sensitivity of intestinal cells to 5-HT, thereby promoting intestinal smooth muscle contraction and peristaltic reflexes [63]. Additionally, downregulated expression of the *sert* gene reduces the reuptake of 5-HT, thus amplifying the physiological effects of 5-HT [64]. Supporting the role of this pathway, the *L. casei* Shirota has also been reported to alleviate constipation by increasing intestinal 5-HT concentrations [65]. Although genomic evidence for 5-HT synthesis in BGI-N2 is lacking, its modulation of intestinal 5-HT levels in zebrafish may achieved indirectly via secondary metabolite regulation or host-microbiota interactions. The specific metabolic pathways necessitate further experimental validation.

This study has several limitations that require further research. First, while genomic analysis provides preliminary support for the safety of BGI-N2, more comprehensive in vivo and in vitro safety assessments are warranted to fully establish its safety profile as a probiotic candidate. Second, whereas the zebrafish model possesses clear advantages for quick screening and initial mechanism studies, it differs significantly from mammals in key aspects, notably the lack of a stomach with a simpler intestinal tract, and a gut microbiota predominantly composed of Proteobacteria [54]. Therefore, further validation in mammalian models is essential to substantiate the potential of BGI-N2 as a promising preclinical candidate. Third, although our findings demonstrate the regulatory role of BGI-N2 on the 5-HT signaling pathway, a more detailed understanding of its upstream trigger mechanism calls for comprehensive analysis using metabolomics and other advanced techniques.

## Conclusion

In conclusion, this work first presents the complete genome sequence of BGI-N2, provides robust scientific support for its development as a novel, effective, and safe prokinetic probiotic candidate, and contributes a promising microbial intervention strategy for constipation.

## Supplementary Information

Supplementary Material 1.

## Data Availability

The dataset supporting the conclusions of this article is available in the China National GenBank Database (CNGBdb): CNP0008109 (https://db.cngb.org/data_resources/project/CNP0008109).

## Abbreviations

BGI-N2: *Lacticaseibacillus paracasei* BGI-N2
PEG: Polyethylene Glycol
SCFAs: Short-Chain Fatty Acids
5-HT: 5-hydroxytryptamine
CGR2: Cultivated Genome Reference 2
LGG: *Lacticaseibacillus rhamnosus* GG
ANI: Average Nucleotide Identity
COG: Clusters of Orthologous Groups
KEGG: Kyoto Encyclopedia of Genes and Genomes
CAZymes: Carbohydrate-Active Enzymes
ARGs: Antibiotic Resistance Genes
VFs: Virulence Factors
RGI: Resistance Gene Identifier
VFDB: Virulence Factor Database
CFS: Cell-Free Supernatant
NC: Normal Control
MOD: Model Control
MTC: Maximum Tolerable Concentration
LD: Low Dose
MD: Medium Dose
HD: High Dose
NO: Nitric Oxide
MTL: Motilin
qPCR: Quantitative real-time PCR
GHs: Glycoside Hydrolases
GTs: Glycosyl Transferases
