# Supplementary material for: Mechanisms of constipation alleviation by Lacticaseibacillus paracasei BGI-N2: insights from genes to phenotypes
Source: BMC Microbiol. 2026 Feb 20;26:280. doi: 10.1186/s12866-026-04831-0 (PMC13032640; doi:10.1186/s12866-026-04831-0)
Supplement: Supplementary file 1 — Supplementary Material 1. [file 12866_2026_4831_MOESM1_ESM.docx]

Table S1 Gene-specific primer sequences for qPCR amplification

| **Gene** | **Primer Sequences** | |
| --- | --- | --- |
| *β-actin* | Forward | TCGAGCAGGAGATGGGAACC |
|  | Reverse | CTCGTGGATACCGCAAGATTC |
| *sert* | Forward | AAAATGGAGGGGGTGCCTTT |
|  | Reverse | GGGAGTGTTCCATCGGTTGT |
| *kitla* | Forward | TGACCCCGAAAAAGGGGCAT |
|  | Reverse | AACAGAGCGGGACCTTCTTC |
| *kitb* | Forward | AAATGCTAAAACCGAGTGCG |
|  | Reverse | CCATCGCCTGTTTTGGAACTG |
| *kitlb* | Forward | CTCCTTTCACGCCAGGTCAA |
|  | Reverse | TCTTGCTCCACAACGACCTC |
| *htr1aa* | Forward | CGGATAAATGCTTGGCGGTG |
|  | Reverse | TGGCAGGCTCAGGTGATTTT |
| *tph1a* | Forward | CTCGGAATGACTTTGGAGGAGA |
|  | Reverse | GCACATTCTCCATCTCGCTTTC |
| *tph1b* | Forward | ACATGCCGCGATTGTCTTCT |
|  | Reverse | GCACCTCAGTAAGATCGCTTG |
| *tph2* | Forward | TGCTCCGCAGGACATCCTTTAAT |
|  | Reverse | ACACAGTCCAAACAGTCGGTCTC |

Table S2 Genes related to antioxidant activity, adhesion, antibacterial activity, and adaptation in the BGI-N2 genome

| **Type** | **Preferred Name** | **EC** | **KEGG** | **PFAMs** |
| --- | --- | --- | --- | --- |
| Antioxidantion | *mntH* | - | K03322 | Nramp |
|  | *trxA* | - | K03671 | Thioredoxin |
|  | *npr* | 1.11.1.1 | K05910 | Pyr_redox_2  Pyr_redox_dim |
|  | *tpx* | 1.11.1.15 | K11065 | AhpC-TSA  Redoxin |
|  | *trxB* | 1.8.1.9 | K00384 | Pyr_redox_2 |
|  | *msrA* | 1.8.4.11 | K07304 | PMSR |
|  | *nrdH* | - | K06191 | Glutaredoxin |
|  | *msrB* | 1.8.4.12 | K07305 | SelR |
|  | *poxL* | 1.2.3.3 | K00158 | TPP_enzyme_C  TPP_enzyme_M  TPP_enzyme_N |
|  | *ndh* | 1.6.99.3 | K03885 | DoxX  Pyr_redox_2 |
|  | *mntA* | - | K19975  K19976 | ZnuA |
|  | *ahpC* | 1.11.1.15 | K03386 | 1. cysPrx_C   AhpC-TSA |
| Antibacterial substance | *alsS* | 2.2.1.6 | K01652 | TPP_enzyme_C  TPP_enzyme_M  TPP_enzyme_N |
|  | *nox* | 1.6.3.4 | K17869 | Pyr_redox_2  Pyr_redox_dim |
|  | *ldh* | 1.1.1.27 | K00016 | Ldh_1_C  Ldh_1_N |
| Adhesion | *bgaC* | 3.2.1.23 | K12308 | BetaGal_dom4_5  Glyco_hydro_35 |
|  | *eno* | 4.2.1.11 | K01689 | Enolase_C  Enolase_N |
|  | *lspA* | 3.4.23.36 | K03101 | Peptidase_A8 |
|  | *mapA* | 2.4.1.8 | K00691 | Glyco_hydro_65C  Glyco_hydro_65N  Glyco_hydro_65m |
|  | *pgi* | 5.3.1.9 | K01810 | PGI |
|  | *tpiA* | 5.3.1.1 | K01803 | TIM |
|  | *tuf* | - | K02358  K15771 | GTP_EFTU  GTP_EFTU_D2  GTP_EFTU_D3 |
| Antestinal adaptability | *cps2E* | - | - | Bac_transf  CoA_binding_3 |
|  | *cps2D* | 5.1.3.2 | K01784 | Epimerase  GDP_Man_Dehyd |
|  | *cps3J* | - | - | DUF4422 |
|  | *cps1C* | - | K03328 | Polysacc_synt  Polysacc_synt_C |
|  | *rpoN* | - | K03092 | Sigma54_AID  Sigma54_CBD  Sigma54_DBD |
|  | *rpoE* | - | K03048 | HARE-HTH |
|  | *luxS* | 4.4.1.21 | K07173 | LuxS |

Table S3 Antibiotic resistance genes identified in BGI-N2 genome

| **RGI Criteria** | **Gene**  **Name** | **AMR Gene**  **Family** | **Drug**  **Class** | **Identify(%)** | **Query**  **Cover (%)** |
| --- | --- | --- | --- | --- | --- |
| Strict | *qacJ* | small multidrug resistance antibiotic efflux pump | disinfecting agents, antiseptics | 38.24 | 99.07% |

Table S4 Virulence factors identified in BGI-N2 genome

| **Subject_ID** | **VF Name** | **VF Category** | **Identity (%)** | **Query Cover (%)** |
| --- | --- | --- | --- | --- |
| VFG002165 | *EfaA* | adherence | 60.99 | 89 |
| VFG006717 | *Lap* | adherence | 61.12 | 98 |
| VFG046465 | *EF-Tu* | adherence | 69.98 | 99 |
| VFG012095 | *GroEL* | adherence | 68.95 | 96 |
| VFG000079 | *ClpC* | stress survival | 61.06 | 97 |
| VFG000080 | *ClpE* | stress survival | 62.28 | 98 |
| VFG000077 | *ClpP* | stress survival | 69.74 | 99 |
| VFG037100 | *MsrAB* | stress survival | 60.15 | 91 |
| VFG000964 | *HA capsule* | immune modulation | 69.80 | 97 |
| VFG002190 | *Capsule* | immune modulation | 63.71 | 98 |
| VFG048830 | *Capsule* | immune modulation | 67.52 | 99 |
| VFG001405 | *SigA* | regulation | 63.04 | 64 |

Table S5 The maximum tolerable toxic concentration of zebrafish to BGI-N2

| **Group** | **Concentration (µg/mL)** | **Viable Count**  **（×10^8^ CFU/mL）** | **Mortality Count** | **Mortality Rate (%)** | **Phenotype** |
| --- | --- | --- | --- | --- | --- |
| NC | - | - | 0 | 0 | No observable abnormalities |
| BGI-N2 | 125 | 0.3294 | 0 | 0 | Comparable to NC group |
|  | 250 | 0.6588 | 0 | 0 |  |
|  | 500 | 1.3175 | 0 | 0 |  |
|  | 1000 | 2.6350 | 0 | 0 |  |
|  | 2000 | 5.2700 | 0 | 0 |  |
